# Molecular profiling of EBV associated diffuse large B-cell lymphoma

**DOI:** 10.1038/s41375-022-01804-w

**Published:** 2023-01-05

**Authors:** Fabian Frontzek, Annette M. Staiger, Ramona Wullenkord, Michael Grau, Myroslav Zapukhlyak, Katrin S. Kurz, Heike Horn, Tabea Erdmann, Falko Fend, Julia Richter, Wolfram Klapper, Peter Lenz, Stephan Hailfinger, Anna Tasidou, Marcel Trautmann, Wolfgang Hartmann, Andreas Rosenwald, Leticia Quintanilla-Martinez, German Ott, Ioannis Anagnostopoulos, Georg Lenz

**Affiliations:** 1 Department of Medicine A, Department of Hematology, Oncology and Pneumology, University Hospital Münster, Münster, Germany; 2 Department of Clinical Pathology, Robert Bosch Hospital, Stuttgart, Germany; 3 Dr. Margarete Fischer-Bosch Institute of Clinical Pharmacology, Stuttgart and University of Tuebingen, Tuebingen, Germany; 4 Institute of Pathology and Neuropathology, Reference Center for Haematopathology University Hospital, Tübingen Eberhard-Karls-University, Tübingen, Germany; 5 Division of Hematophathology, Christian-Albrechts-University, Kiel, Germany; 6 Department of Physics, University of Marburg, Marburg, Germany; 7 Department of Hematopathology, Evangelismos General Hospital, Athens, Greece; 8 Division of Translational Pathology, Gerhard-Domagk-Institute of Pathology, University Hospital Münster, Münster, Germany; 9 Institute of Pathology, University of Würzburg, Würzburg, Germany

**Keywords:** Translational research, Cancer genomics

## Abstract

Epstein-Barr virus (EBV) associated diffuse large B-cell lymphoma (DLBCL) represents a rare aggressive B-cell lymphoma subtype characterized by an adverse clinical outcome. EBV infection of lymphoma cells has been associated with different lymphoma subtypes while the precise role of EBV in lymphomagenesis and specific molecular characteristics of these lymphomas remain elusive. To further unravel the biology of EBV associated DLBCL, we present a comprehensive molecular analysis of overall 60 primary EBV positive (EBV+) DLBCLs using targeted sequencing of cancer candidate genes (CCGs) and genome-wide determination of recurrent somatic copy number alterations (SCNAs) in 46 cases, respectively. Applying the LymphGen classifier 2.0, we found that less than 20% of primary EBV + DLBCLs correspond to one of the established molecular DLBCL subtypes underscoring the unique biology of this entity. We have identified recurrent mutations activating the oncogenic JAK-STAT and NOTCH pathways as well as frequent amplifications of 9p24.1 contributing to immune escape by PD-L1 overexpression. Our findings enable further functional preclinical and clinical studies exploring the therapeutic potential of targeting these aberrations in patients with EBV + DLBCL to improve outcome.

## Introduction

Epstein-Barr virus (EBV) belongs to the family of γ-herpesviruses with the vast majority of the world population being infected by EBV once during lifetime [1]. After active infection, EBV persists quiescently in memory B-cells. Although EBV frequently remains a harmless passenger virus, EBV reactivation is a known factor to immortalize B-cells and to promote lymphomagenesis in a subset of individuals [2]. EBV infection of lymphoma cells has been associated with various specific lymphoma subtypes such as Burkitt, classic Hodgkin (cHL), plasmablastic, pleural effusion, extranodal natural killer (NK)/T-cell nasal type, or diffuse large B-cell lymphoma (DLBCL) [3]. However, the precise molecular mechanisms how EBV infection impacts the biology of distinct lymphoma subtypes remain elusive.

EBV positive (EBV+) DLBCL is a rare aggressive B-cell lymphoma subtype with the majority of lymphoma cells being infected by EBV [3]. In Western countries less than 5% of DLBCLs can be classified as EBV + DLBCL [4, 5] although precise incidences in different geographic regions remain unclear, especially as inconsistent cut-offs to determine positivity of EBV-encoded small RNAs (EBER) have been used in previous studies [6]. In line with the age distribution of DLBCL NOS, EBV + DLBCL predominantly affects elderly patients but is also found in younger individuals [5, 7]. Underlying immunodeficiency and/ or immunosuppression as well as a history of prior lymphoma have to be excluded for diagnosis [3]. EBV + DLBCLs share several features with post-transplant lymphoproliferative disorders (PTLD) in terms of morphology and EBV latency programs [6]. In retrospective studies, EBV + DLBCLs have been associated with an aggressive clinical course leading to adverse overall survival of affected patients [8, 9].

Insights into the molecular pathogenesis of EBV + DLBCLs remain scarce since available studies have mainly characterized small cohorts of patients [10–12]. Recently, recurrent mutations of components of the JAK-STAT, WNT, and NF-κB signaling pathways have been reported [10, 11]. However, additional studies are required to fully uncover underlying molecular mechanisms and to potentially derive novel therapeutic strategies for affected patients. To this end, we performed a comprehensive molecular analysis combining targeted sequencing, genome-wide copy number analysis as well as the determination of the molecular subtypes in a cohort of 60 primary EBV + DLBCL samples. For the first time, we reveal that less than 20% of primary EBV + DLBCLs can be attributed to previously described molecular subtypes of DLBCL. Here, we describe recurrent targetable genetic aberrations leading to activation of JAK-STAT and NOTCH signaling, perturbation of epigenetic regulators as well as distinct genetic lesions contributing to the immune evasion of lymphoma cells.

## Methods

### Patient samples

We collected primary formalin-fixed and paraffin-embedded (FFPE) samples of patients with the diagnosis of EBV + DLBCL from the University Hospitals in Münster, Kiel, Würzburg, Tübingen, Berlin, and the Robert-Bosch-Hospital in Stuttgart. To confirm the diagnosis of EBV + DLBCL, all samples were centrally and independently reevaluated by three expert hematopathologists (German Ott, Ioannis Anagnostopoulos, Andreas Rosenwald). All specimens had to display large cell morphology and were to express CD20 and EBER by the neoplastic cells. From initially 76 cases, 60 cases were finally included in the study (Supplementary Fig. 1, Supplementary Table 1). For all included cases, no state of immunodeficiency including HIV infection, history of prior lymphomas, or iatrogenic immunosuppression were reported. Our study was reviewed and approved by the ethical review board of the University Hospital Tübingen in accordance with the Declaration of Helsinki. All data were fully anonymized.

### Immunohistochemistry and fluorescence in situ hybridization

Immunohistochemical stainings and EBER in situ hybridization were performed according to standard protocols as previously described [13, 14]. Primary EBV + DLBCL cases were uniformly stained with anti-PD-L1 (CD 274) antibody (clone SP142, Roche, Basel, Switzerland). Cases with more than 10% tumor cells expressing PD-L1 were scored positive.

Fluorescence in situ hybridization (FISH) was performed as previously described [15, 16]. Specimens were hybridized with the Vysis LSI *MYC* dual color break-apart probe (BAP; Abbott Molecular, Wiesbaden, Germany), Vysis LSI *BCL2* Dual Color BAP (Abbott), and Vysis LSI *BCL6* Dual Color BAP (Abbott) (Supplementary Table 1). The scoring was performed according to standard procedures [17].

### Extraction of RNA and DNA

DNA and RNA were extracted from FFPE specimens using the AllPrep DNA/ RNA FFPE KIT 50 (Qiagen, Hilden, Germany). Concentrations were determined by fluorometer using Qubit 1X dsDNA HS Assay Kit (Thermo Fisher Scientific, Waltham, Massachusetts, USA) and Qubit RNA HS Assay Kit (Thermo Fisher Scientific), respectively.

### Determination of cell-of-origin

To determine the molecular subtype according to the cell-of-origin (COO) classification, extracted RNA was analyzed by the NanoString nCounter FLEX gene expression profiling (GEP) system (NanoString, Seattle, Washington, USA) as previously described [18]. The NanoString Lymphoma Subtyping Test (LST) algorithm allows the assignment of each analyzed sample to the germinal center B-cell like (GCB) subtype, the activated B-cell like (ABC) subtype, or to be unclassified (Supplementary Table 1) [19, 20]. In brief, the LST CodeSet consists of capture and reporter probes for 20 genes: 7 genes overexpressed in GCB DLBCL (*ASB13, ITPKB, MAML3, MME, MYBL1, S1PR2, SERPINA9*), 8 genes overexpressed in ABC DLBCL (*CCDC50, CREB3L2, CYB5R2, IRF4, LIMD1, PIM2, RAB7L1, TNFRSF13B*), and 5 housekeeping genes (*ISY1, R3HDM1, TRIM56, UBXN4, WDR55*). Quality and quantity of used RNA was determined by spectrophotometer (Nanodrop, Thermo Scientific). The LST CodeSet was hybridized to 500 ng of total RNA for 18 h at 65 °C. Hybridized RNA samples were loaded into the nCounter Prep Station and expression of target mRNA was finally assessed by the nCounter Digital Analyzer.

### Targeted sequencing and analysis of somatic DNA mutations

We extracted 200 ng DNA per sample for targeted sequencing. Targeted deep sequencing was performed for 74 genes that were previously identified to be recurrently mutated in DLBCL (Supplementary Table 2). Sequencing was analyzed on a HiSeq platform (Illumina, San Diego, California, USA) with 250 bp paired-end reads. We achieved a median effective coverage of 341 reads per gene. An overview of our analysis pipeline with all integrated key methods and utilized external databases is depicted in Supplementary Fig. 2. All steps are explained in detail in the Supplementary Methods. All filter steps in the applied order are listed in Supplementary Table 3 and all somatic mutations are provided in Supplementary Table 4. To further identify putative CCGs, we applied the cancer gene prediction algorithm dNdScv to our dataset [21]. Notably, dNdScv relies on information regarding synonymous variants that is limited in the context of targeted sequencing, hence its detection precision is restricted in comparison to whole exome sequencing.

### Analysis of somatic copy number alterations (SCNAs)

To discover genome-wide SCNAs, 80 ng of DNA were extracted and subsequently analyzed using the OncoScan CNV FFPE Assay Kit (Affymetrix, Thermo Fisher Scientific) as previously described following the manufacturer’s instructions [22].

Raw data were preprocessed according to manufacturer instructions (Chromosome Analysis Suite 4.3 (ChAS) Software, Thermo Fisher Scientific). For allele-specific copy number segmentation at sample level, we applied ASCAT v2.4.2 [23] (Supplementary Table 5). ASCAT estimates sample ploidy and purity (i.e. the cell fraction originating from aberrant tumor cells, as opposed to non-aberrant bystander cells). Recurrent SCNAs at the cohort level were identified and statistically evaluated using GISTIC 2.0 [24] (Supplementary Table 6).

### Determination of molecular DLBCL clusters

To assign samples to previously described genetic DLBCL subtypes, we applied the LymphGen 2.0 classifier (https://llmpp.nih.gov/lymphgen/lymphgendataportal.php) to all available measured data comprising recurrent mutations, SCNAs, and translocation status of *BCL2* and *BCL6* determined by FISH [25, 26] (Supplementary Table 1). We aimed to identify the previously defined genetic DLBCL clusters A53, BN2, EZB, MCD, N1, and ST2.

### Statistical association analyses

We analyzed associations of gene mutations and SCNAs with the following defined subgroups: samples of ABC *vs*. GCB DLBCL subtype, ABC DLBCL subtype *vs*. unclassified, ABC *vs*. non-ABC DLBCL subtype, GCB subtype *vs*. unclassified DLBCL, and GCB vs. non-GCB DLBCL subtype. To test for a significantly higher median mutation or SCNA count in one subcohort *vs*. the other, we utilized a one-tailed Wilcoxon rank-sum test for each selected genetic lesion (Supplementary Table 7). Our hypotheses were focused on recurrently mutated genes with cohort frequency ≥7% (Supplementary Table 4) and all recurrent SCNAs reaching significance according to GISTIC (cohort frequency ≥5%, q_G2.0_ < 0.1, Supplementary Table 6). False discovery rates (FDR) were calculated using the Benjamini and Hochberg (BH) method [27]. We applied the prescribed error threshold of *q* < 0.1 for significance.

## Results

### Study cohort, *MYC* translocation status, and cell-of-origin

In total, 51 primary EBV + DLBCL FFPE samples fulfilled the diagnostic criteria and showed more than 50% of lymphoma cells with positive EBER staining. As no clear cut-off for EBER staining has been established in the literature so far and variable cut-offs have been applied in previous studies [6], we added 9 further primary DLBCL cases displaying positive EBER in less than 50% of cells (range: 10–40%, median: 30%) to our study cohort (Supplementary Table 1).

The median age of included patients was 69 years (range: 26–91) with 7 patients (12%) being younger than 50 years. 38 of 60 patients (63%) were male (Supplementary Table 1). No state of immunodeficiency, history of other lymphomas, or iatrogenic immunosuppression were reported in any of the patients.

In total, 23 primary EBV + DLBCL FFPE samples with sufficient tissue could be analyzed by FISH applying an *MYC*-break apart probe. Two out of 23 cases (9%) showed an *MYC* translocation corresponding to the frequency reported for DLBCL not otherwise specified (NOS) [28]. One single case with *MYC* translocation harbored an additional *BCL6* translocation (double hit), whereas both *MYC* translocated cases were negative for a concomitant *BCL2* translocation.

**Fig. 1:**
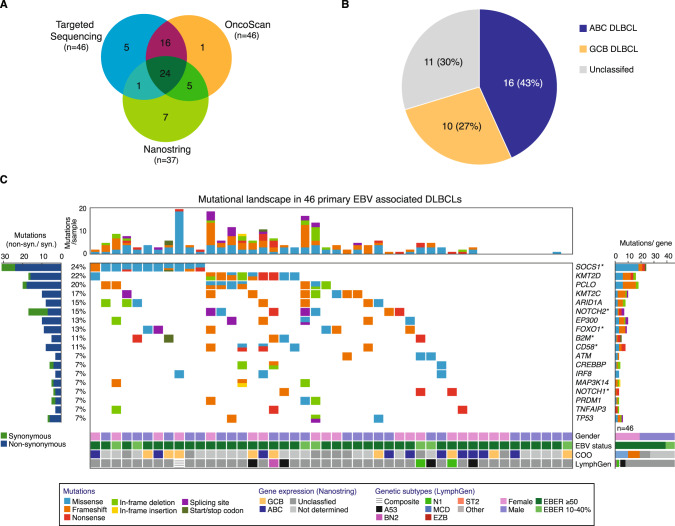
Mutational landscape. **A** Venn diagram illustrating number of samples analyzed by targeted sequencing (*n* = 46), OncoScan (*n* = 46), and NanoString (*n* = 37), respectively. **B** Pie chart showing the distribution of 37 primary EBV + DLBCLs with respect to ABC DLBCL (*n* = 16), GCB DLBCL (*n* = 10), or unclassifiable DLBCL (*n* = 11) based on NanoString nCounter FLEX gene expression profiling. **C** All detected non-synonymous mutations are depicted for each primary EBV + DLBCL sample per column (*n* = 46) and are sorted by cohort frequency (see Supplementary Table 4 for all results). Sample order is based on waterfall sorting by binary gene mutation status. Genes reaching significance according to dNdScv (q < 0.1) are highlighted (*). Bar graphs at the left show the ratio of non-synonymous (blue)/ synonymous mutations (green) detected per gene. Bar graphs at the top depict the number of called mutations per sample. Detected mutations per gene are shown at the right. Type of mutations are color-coded. Gender, EBV status (EBER > 50% or EBER 10–40%), cell of origin (COO), and genetic subtype according to the LymphGen classifier are indicated for each sample.

To determine the cell-of-origin, we performed NanoString analyses for 37 primary EBV + DLBCLs for which we were able to extract sufficient amounts of RNA (Supplementary Table 1, Fig. 1A). Overall, 16 cases (43%) were attributed to the ABC subtype, 10 cases (27%) were classified as GCB DLBCL, and 11 cases (30%) remained unclassified (Fig. 1B).

### Targeted sequencing of selected cancer candidate genes in EBV+DLBCLs

To explore the mutational landscape of EBV + DLBCLs, we applied targeted sequencing of CCGs in a cohort of 46 primary EBV + DLBCL samples (Fig. 1C, Supplementary Tables 3–4). The panel of target genes comprised 74 selected cancer genes recurrently mutated in DLBCL and other lymphoid malignancies (Supplementary Table 2). Six genes reached significance according to dNdScv (*q* < 0.1) and were highlighted in Fig. 1C (Supplementary Table 4).

**Fig. 2:**
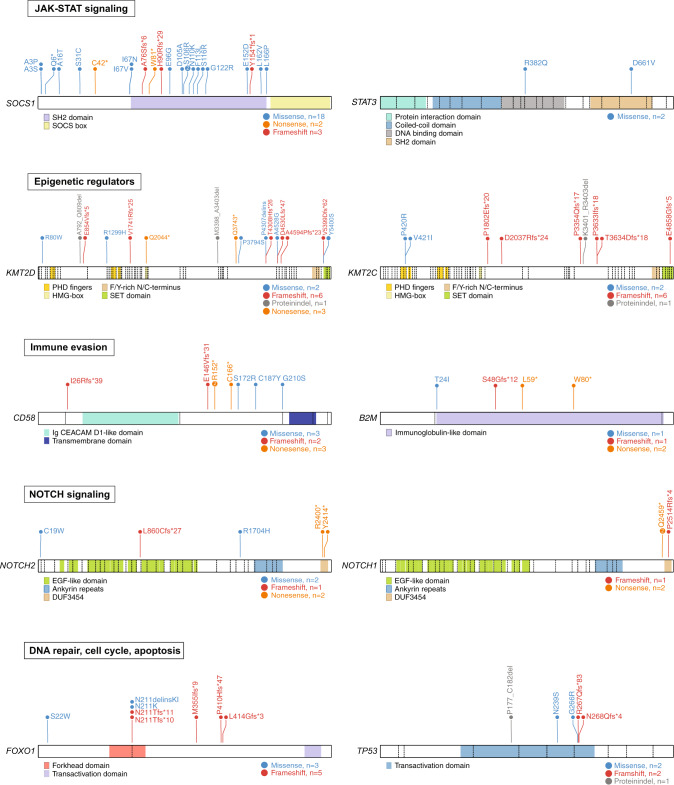
Recurrent mutations on protein level. Needle plots illustrating the distribution of detected mutations on protein level for the selected cancer candidate genes and their particular isoforms *SOCS1* (NM_003745.1), *STAT3* (NM_139276), *KMT2D* (NM_003482.3), *KMT2C* (NM_170606.2), *CD58* (NM_001779.2), *B2M* (NM_004048.2), *NOTCH2* (NM_024408.3), *NOTCH1* (NM_017617.4), *FOXO1* (NM_002015.3), and *TP53* (NM_001126112.2). Selected protein domains are shown in color and types of mutation are indicated. Exon boundaries are visualized by dashed lines.

**Fig. 3:**
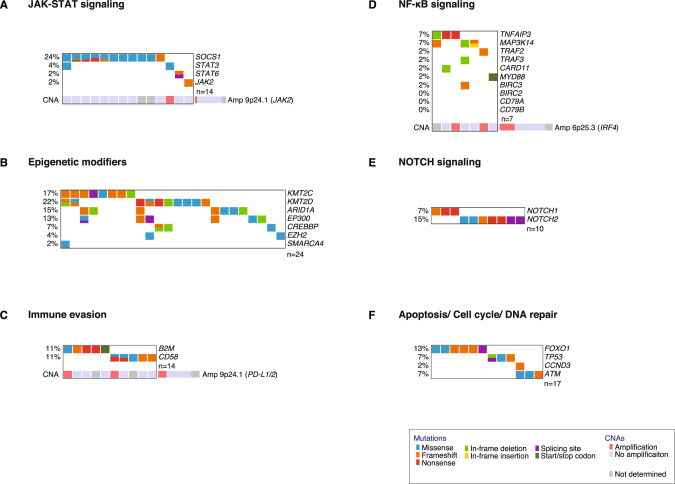
Co-occurrence of selected mutations and SCNAs affecting the same biological pathways. Alterations belonging to one sample are shown per column. Sample order is based on waterfall sorting by binary gene mutation status.

The most frequently mutated gene was *SOCS1* in 24% of cases reaching significance according to dNdScv (*p* = 0.0008, *q* = 0.02, Supplementary Table 4, Fig. 1C). SOCS1 is a negative regulator of the JAK-STAT and interferon gamma (INFγ) signaling pathways [29, 30]. Roughly two thirds (14/23, 61%) of detected *SOCS1* mutations were located in the SH2 domain that mediates binding of SOCS1 to the Janus kinases (JAK) (Fig. 2) [29]. Six out of 11 (55%) *SOCS1* mutated samples harbored multiple *SOCS1* mutations further underscoring that they represent loss of function aberrations. Additional mutations affecting genes encoding components of the JAK-STAT pathway were detectable in *STAT3*, *STAT6*, and in *JAK2* (Figs. 2, and 3A). Thus, overall, 30% (14/46) of analyzed primary EBV + DLBCLs harbored mutations affecting this oncogenic pathway (Fig. 3A).

The genes encoding the lysine methyltransferases KMT2D and KMT2C were mutated in 22% and 17% of cases, respectively (Fig. 1C). The genes encoding other important epigenetic regulators including ARID1A, EP300, and CREBBP were mutated in 15%, 13%, and 7% of cases, respectively. Overall, more than the half of cases (24/46, 52%) were affected by mutations of genes encoding chromatin modifiers (Fig. 3B).

A further hallmark of EBV associated DLBCLs represents molecular mechanisms contributing to immune evasion. Mutations affecting *CD58* and *B2M* were detectable in 11% of cases, respectively (Fig. 1C). Both genes were detected as significant drivers according to dNdScv (Supplementary Table 4). Five out of eight detected *CD58* mutations were frameshift or nonsense aberrations (Fig. 2). Mutations of *CD58* and *B2M* were mutually exclusive (Fig. 3C).

The most frequently mutated genes involved in NF-κB signaling were *TNFAIP3* encoding A20 and *MAP3K14* encoding NIK in 7%, respectively (Fig. 3D). Frequent mutations occurring in DLBCL NOS such as *CD79A/B*, *CARD11*, or *MYD88* aberrations occurred at low frequency or were completely absent (Fig. 3D).

In total, 22% of primary EBV + DLBCLs (10/46) harbored mutations affecting *NOTCH1* or *NOTCH2*. dNdScv confirmed both genes as significant cancer driver genes (Supplementary Table 4). Both mutations occurred in a mutually exclusive fashion (Fig. 3E). All three detected *NOTCH1* mutations clustered in the C-terminal proline, glutamic acid, serine, and threonine (PEST) degron domain and were nonsense or frameshift mutations (Fig. 2). Inactivation of the PEST degron domain is a known mechanism to enhance NOTCH signaling [31, 32]. Two out of five detected *NOTCH2* mutations were located in the C-terminal PEST domain (Fig. 2).

Finally, different tumor suppressor genes were affected by recurrent alterations (Fig. 3F). Mutations of *FOXO1* were detectable in 13% of cases and reached significance according to dNdScv (*p* = 0.006, *q* = 0.06, Supplementary Table 4). We uncovered a mutational hotspot at codon N211 within the forkhead DNA binding domain (4/8, 50%) (Fig. 2). Mutations located within the forkhead DNA binding domain might be able to disrupt the binding and the subsequent stabilization by 14-3-3 [33, 34]. Mutations of the tumor suppressor gene *TP53* were detected in 7% of cases and exclusively affected the transactivation domain (Fig. 2).

We did not find any significant associations of recurrent mutations and COO classification determined by NanoString (Supplementary Table 7).

### Genome-wide analysis of copy number alterations in EBV+DLBCLs

**Fig. 4:**
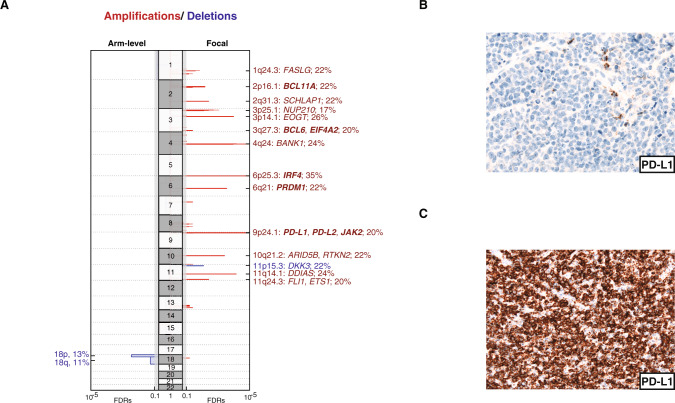
Recurrent copy number alterations. **A** GISTIC v2.0.23 defined copy number amplifications (red) and deletions (blue) are visualized for each chromosome, arm-level alterations on the left, and focal lesions on the right. Insignificant lesions (*q*_G2.0_  >  0.1) are shaded in gray. Selected potential driver genes are shown with corresponding cohort frequencies (cosmic cancer genes are shown in bold letters). **B**, **C** Representative images of immunohistochemical PD-L1 stainings of a PD-L1 negative (**B**) and a PD-L1 positive (**C**) EBV + DLBCL case.

For the first time, we present a comprehensive genome-wide analysis of recurrent SCNAs in a cohort of 46 primary EBV associated DLBCLs (Fig. 4A, Supplementary Tables 5–6). To this end, we used the OncoScan CNV FFPE Assay and subsequently used the GISTIC v2.0.23 algorithm [24].

Six cases (13%) were polyploid as determined by ASCAT [23]. We did not find any broad arm-level but various focal amplifications (Fig. 4A). The most significant one was an amplification affecting 6p25.3 detected in 35% of cases (*q* = 7.5*10^−7^). Within this circumscribed aberration, only 5 different genes including the oncogene *IRF4* are located. In 20% of primary EBV associated DLBCLs, focal amplifications of 9p24.1 comprising *PD-L1/-L2* and *JAK2* were detectable (*q* = 1.0*10^−6^). To further correlate *PD-L1* gene amplifications and PD-L1 expression on protein level, we performed uniform immunohistochemical staining of PD-L1 in 24 evaluable FFPE specimens (Fig. 4B, C). Nine out of these 24 cases harbored a *PD-L1* copy gain or amplification (Table 1). Eight out of the nine cases with the *PD-L1* amplification showed strong PD-L1 expression by immunohistochemistry. One single case harboring a *PD-L1* amplification was scored to be negative in immunohistochemistry although focal clusters of clearly positive lymphoma cells were detectable in less than 10% of cells. Out of 15 cases without *PD-L1* amplification, 3 cases (20%) showed PD-L1 expression suggesting that gene amplification represents the major but not the only molecular mechanism leading to PD-L1 protein expression in EBV + DLBCLs (*p* = 0.002, Fisher exact test).

**Table 1:** Correlation of *PD-L1* amplification and protein expression.

Sample ID	Copy number of *PD-L1* locus	Immunhistochemical staining
Ly27	Amplification	Positive
Ly34	Amplification	Positive
Ly55	Amplification	Positive
Ly81	Amplification	Positive
Ly83	Amplification	Positive
Ly21	Gain	Positive
Ly31	Gain	Positive
Ly64	Gain	Positive
Ly36	Wildtype	Positive
Ly37	Wildtype	Positive
Ly49	Wildtype	Positive
Ly4	Amplification	Negative
Ly32	Wildtype	Negative
Ly33	Wildtype	Negative
Ly35	Wildtype	Negative*
Ly39	Wildtype	Negative
Ly40	Wildtype	Negative
Ly42	Wildtype	Negative
Ly44	Wildtype	Negative
Ly45	Wildtype	Negative*
Ly50	Wildtype	Negative
Ly51	Wildtype	Negative*
Ly53	Wildtype	Negative*
Ly54	Wildtype	Negative

^*^Background enriched in inflammatory cells.

In 22% of analyzed cases we identified focal amplifications affecting 1q24.3 as a potentially additional genetic lesion contributing to immune escape. Specifically, amplification of the *FAS ligand* (*FASL*) (*q* = 0.027) located at the locus 1q24.3 might enable lymphoma cells to induce apoptosis of surrounding lymphocytes [35].

We additionally uncovered focal amplifications of 11q24.3 affecting the oncogenic transcription factors *ETS1* and *FLI1* in 20% of EBV + DLBCLs (*q* = 0.0038). Finally, we detected circumscribed focal amplifications of 2q31.3 containing only three different genes (*q* = 0.0038). Within this aberration, we identified the long non-coding RNA *SChLAP1* that was reported to be overexpressed in a subset of prostate cancers characterized by aggressive clinical behavior [36].

Overall, deletions occurred less frequently compared to amplifications. Broad arm level deletions of 18p and 18q occurred in 13% and 11%, respectively. The only significant focal deletion affected the chromosomal locus 11p15.3 which was detectable in 22% of cases (*q* = 0.0085). Among recurrently deleted genes within this aberration, we identified *DKK3* as tumor suppressor gene and potential inhibitor of WNT signaling [37].

Detected significant SCNAs were not associated with specific gene expression profiles determined by NanoString (Supplementary Table 7).

### Molecular clusters in EBV+DLBCLs

**Fig. 5:**
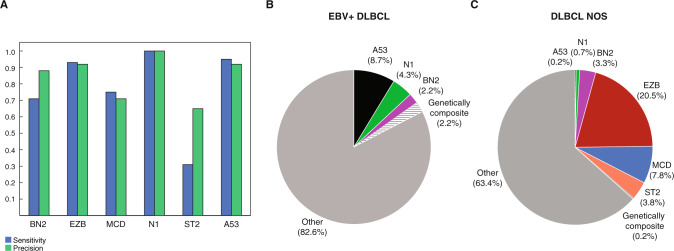
Molecular DLBCL clusters. **A** Bar graph showing the sensitivity (blue) and precision (green) for prediction of molecular clusters in our cohort of EBV + DLBCLs applying the Full Model according to the LymphGen classifier 2.0. **B** Pie chart depicting the distribution of molecular subtypes in our cohort of EBV + DLBCLs (*n* = 46) and (**C**) in a cohort of DLBCLs NOS (*n* = 839) according to the LymphGen classifier 2.0 [25, 38].

In an integrative analysis, we combined the available data of recurrent mutations, SCNAs, and structural variants affecting *BCL2* and *BCL6* in order to attribute each EBV + DLBCL sample to one of the defined molecular DLBCL clusters A53, N1, BN2, EZB, and MCD applying the LymphGen 2.0 classifier [25, 26]. All 46 samples with available sequencing data were included in this analysis. For 40 out of these 46 samples, copy number analyses were performed. For 32 and 30 out of these 46 samples (70%/ 65%), we were able to investigate the *BCL2* and *BCL6* translocation status by FISH, respectively. Overall, only one single case harbored a *BCL2* (3%) and two cases a *BCL6* translocation (7%). Applying the “Full Model” of the LymphGen classifier, we reached an overall sensitivity of 71%, 93%, 75%, 100%, 31%, and 95% for the clusters BN2, EZB, MCD, N1, ST2, and A53, respectively (Fig. 5A, Supplementary Table 1, Supplementary Fig. 3). Overall, four cases (8.7%) were attributed to the A53 subtype, two cases (4.3%) to the N1, and one case (2.2%) was identified to belong to the BN2 and the “Genetically composite” subgroups, respectively (Fig. 5B). None of the cases was attributed to the MCD subgroup. Notably, 82.6% of all analyzed cases (*n* = 38) were classified to be “Other” and could not be identified as a specific molecular DLBCL subtype. For comparison, 43% of cases were not assigned to any specific genetic cluster in the LymphGen learning cohort of primary DLBCL NOS cases [25].

To further support that the rather high rate of unclassifiable primary EBV + DLBCLs was related to the distinct biology of disease and not to lacking sensitivity of our analysis, we next applied the LymphGen classifier to a large cohort of 839 primary DLBCLs NOS previously published by Lacy et al. [38]. First, we used all available gene mutations determined by targeted sequencing of 293 genes. Overall, 19.7% of primary DLBCL NOS cases were attributed to the EZB cluster, 8.9% to the ST2 cluster, 5.8% to MCD, 6.3% were classified as BN2, 0.7% as N1, and 0.2% of cases were finally attributed to the molecular clusters A53 and “Genetically composite”, respectively. Thus, 58.1% of all cases were classified to be “Other” and could not be identified as a specific molecular DLBCL subtype. Next, we applied the LymphGen classifier to the restricted panel of genes also applied to our cohort (*n* = 61 overlapping genes). Using the restricted gene panel, we received a very similar distribution of molecular DLBCL clusters: the frequency of ST2 (8.9% vs. 3.8%) and BN2 clusters (6.3% vs. 3.3%) were slightly lower applying the whole vs. restricted gene panel. Overall, 63.4% of DLBCL NOS cases were attributed to “Other” (Fig. 5C). Although information about structural variants were not available for the DLBCL cohort of Lacy et al., information about recurrent mutations was sufficient to assign 36.6% of DLBCLs NOS to a distinct molecular DLBCL cluster in comparison to only 17.4% of our cohort of EBV + DLBCLs for that we also considered genome-wide CNAs and structural variants of *BCL2* and *BCL6* (*p* = 0.007, two tailed Fisher exact test). This analysis further underscores that the high rate of primary EBV + DLBCLs that cannot be assigned to any distinct molecular DLBCL cluster is not due to the restricted targeted gene panel applied in our study, but is most likely caused by the different landscape of genetic aberrations of EBV + DLBCL.

## Discussion

Our study investigating 60 primary EBV + DLBCLs revealed recurrent genetic abnormalities that strongly differ in their frequency from DLBCL NOS and therefore further support the notion that EBV + DLBCL represents a distinct entity. Several previous comprehensive genetic analyses have revealed distinct molecular DLBCL subtypes based on the presence of specific recurrent mutations, SCNAs, and structural variants [26, 39]. Interestingly, applying the LymphGen 2.0 classifier to our data, less than 20% of primary EBV + DLBCLs were attributed to one of these molecular DLBCL subgroups [25]. Although we have not determined mutations genome-wide in our study, we reached a high sensitivity and precision for prediction suggesting that these differences are real. Moreover, applying the same gene panel used in this study to a large cohort of primary DLBCLs NOS shows that indeed a significantly higher fraction of cases can be attributed to a genetic DLBCL cluster. In line, none of our primary EBV + DLBCL cases was attributed to the MCD subgroup that is characterized by concomitant *MYD88* and *CD79B* mutations [25, 26]. These results suggest other molecular mechanisms driving oncogenic NF-κB in EBV + DLBCL, as previous analyses indicated that EBV + DLBCLs are characterized by activation of NF-κB signaling [4, 40]. EBV infection itself has been reported to induce NF-κB signaling in lymphoma cells by overexpressing the latent membrane protein 1 (LMP-1). Specifically, LMP-1 mimics the CD40 ligand leading to constitutive activation of NF-κB [41, 42]. More than 90% of EBV + DLBCLs express LMP-1 investigated by immunohistochemistry [43]. Therefore, it is tempting to speculate that EBV associated DLBCLs do not depend on additional genetic aberrations activating NF-κB due to intrinsic EBV-driven NF-κB signaling. In line with our results, previous reports have shown a low frequency of MCD related mutations [10, 44, 45] (Supplementary Table 9). This observation might also indicate that EBV infection represents an earlier pathogenetic event impacting on the acquisition of additional genetic hits.

Interestingly, it has been previously shown that viral LMP-1 is not only able to induce NF-κB but also to enhance oncogenic JAK-STAT signaling in EBV-infected B-cells [46]. LMP-1 directly interacts with JAK3 leading to downstream activation of STAT proteins [46]. In contrast to NF-κB signaling, where additional activating mutations were scarce, we detected deleterious *SOCS1* mutations in 24% of primary EBV + DLBCLs. SOCS1 represents a key inhibitor of JAK-STAT signaling and inactivating *SOCS1* mutations have been reported in subsets of B-cell lymphomas including EBV-associated DLBCLs in HIV-infected individuals [47, 48]. In EBV associated DLBCLs, *SOCS1* mutations might act synergistically with EBV-driven JAK-STAT activation. Corresponding to our findings, Sarkozy et al. reported recurrent mutations of *STAT3* and *SOCS1* occurring in 15% of polymorphic EBV + DLBCLs [10] (Supplementary Table 9). As constitutive JAK-STAT signaling can be exploited therapeutically, future studies should address the potential of JAK-STAT inhibition in patients suffering from EBV + DLBCL.

Among the most frequently mutated CCGs in primary EBV + DLBCLs, we detected recurrent mutations of *NOTCH2* and *NOTCH1*. *NOTCH* mutations frequently occur in splenic marginal zone lymphoma and chronic lymphocytic leukemia but were also reported in subsets of DLBCLs NOS and EBV + DLBCLs [10, 11, 49–51]. Upon binding of specific ligands, the NOTCH receptor is proteolytically cleaved releasing the NOTCH intracellular domain (NICD) to transduce further signaling. The PEST degron domain of the NOTCH receptor normally leads to the proteolytic degradation of NICD so that PEST inactivating mutations finally transduce NOTCH signaling [31, 32]. Accordingly, most detected *NOTCH* mutations truncated the PEST degron domain probably enhancing NOTCH signaling in primary EBV + DLBCLs. The precise role of *NOTCH2* mutations detected outside of the PEST degron domain remains to be elucidated in future studies. As previously reported for mantle cell lymphoma [51], *NOTCH1* and *NOTCH2* mutations occurred mutually exclusively suggesting a similar oncogenic advantage for affected lymphoma cells. Intriguingly, the viral protein EBV nuclear antigen 2 (EBNA2) and NOTCH share downstream signaling by interaction with the transcription factor RBP-Jκ [52, 53]. Since only a subset of EBV + DLBCLs still express EBNA2 [43], the acquisition of *NOTCH* activating mutations might thus be a compensatory mechanism. To what extent pharmacological NOTCH inhibition can improve current therapeutic approaches for patients with EBV + DLBCL needs further investigation.

Following initial infection, EBV persists mainly in resting memory B-cells and can reactivate by switching latency programs [1]. Especially in immunocompetent individuals, mechanisms to evade the host’s immune system seem to be of major importance. However, EBV + DLBCLs affect, with the possible exception of immune senescence, per definition patients with an intact immune system. Interestingly, we and previous groups uncovered several genetic aberrations contributing to immune evasion of lymphoma cells [10, 11, 44]. In our analysis *CD58* and *B2M* mutations occurred in 11% of cases, respectively. However, these aberrations are not specific for EBV + DLBCL, as genetic aberrations affecting *CD58* and *B2M* have previously been detected in other DLBCL subtypes as well [54]. Since EBV infection of intact B-cells was reported to trigger the presentation of various potential tumor associated antigens contributing to an anti-tumor immunity, mutations targeting the antigen presentation machinery might be an essential step in EBV-driven lymphoma- and carcinogenesis [55–57].

In a genome-wide analysis of recurrent SCNAs, we showed that gain or amplification of 9p24.1 occurred in 20% of primary EBV + DLBCLs. The locus 9p24.1 contains the oncogenes *JAK2* and the immune checkpoint proteins *PD-L1/2*. Especially in nodular sclerosing cHL and primary mediastinal large B-cell lymphoma amplification of 9p24.1 has been identified as genetic key alteration contributing to escape from immune surveillance [58]. Although in cHL 9p24.1 and EBV infection have been described to occur mutually exclusively [59], we show that amplification of 9p24.1 represents the major molecular mechanism contributing to PD-L1 overexpression in primary EBV + DLBCLs. Kataoka et al. showed that not only EBV + DLBCLs but also other EBV-positive lymphomas may frequently harbor aberrations involving the programmed death ligands [44] and even in EBV-driven solid tumors an increased expression of immune checkpoints has been reported [60]. Therefore, targeting the PD-1/ PD-L1 axis therapeutically may represent a promising strategy and the clinical potential of immunotherapy should be further explored.

This study has several limitations since it is of retrospective design, comprises only a limited panel of target genes, and analyzes still a limited number of primary EBV + DLBCL samples which is due to the rareness of disease. Moreover, not all samples were profiled with all technologies due to lack of adequate material and clinical data of included patients could not be retrieved. Although our results are mainly in line with previous analyzes, existing differences might be explained by the underlining heterogeneity of the disease. Significantly larger study cohorts are required to further unravel this heterogeneity. However, our findings illustrate that EBV + DLBCLs show a unique biology with distinct molecular aberrations. Even if similar oncogenic pathways are activated in comparison to DLBCL NOS, the activating genetic mechanisms are significantly different. EBV infection seems to shape the genetic backbone of lymphoma cells giving further evidence that EBV can be more than a passenger virus but a vital co-factor promoting lymphomagenesis. Our analysis motivates further studies focusing on therapeutic strategies targeting NF-κB, JAK-STAT, NOTCH signaling, and the PD-1/ PD-L1 axis in patients with EBV + DLBCL.

## Supplementary information

Supplement Data

Supplementary Table 1

Supplementary Table 3

Supplementary Table 4

Supplementary Table 5

Supplementary Table 6

Supplementary Table 7

## Data Availability

Data generated in this study have been deposited in the European Genome-phenome Archive (EGA) under study accession EGAS00001006400. The targeted sequencing data is available in FASTQ.gz format under the dataset accession EGAD00001009396 ; SNP array raw data in CEL format will still be provided. These data are available under restricted access for German data privacy laws. Access can be obtained via the associated data access committee EGAC00001002755. EGA access will be granted after a data access contract has been agreed with the Department of law of the University Hospital Münster. The processed somatic mutations and copy number alterations as well as clinical metadata are provided in the Supplementary Datasets.

## References

[1] Cohen JI (2000). Epstein-Barr virus infection. N Engl J Med.

[2] Kuppers R (2003). B cells under influence: transformation of B cells by Epstein-Barr virus. Nat Rev Immunol.

[3] Swerdlow SCE; Harris NL; Jaffe ES; Pileri SA; Stein H; Thiele J WHO Classification of tumours of haematopoietic and lymphoid tissues. Revised Fourth Edition. 2017.

[4] Ok CY, Li L, Xu-Monette ZY, Visco C, Tzankov A, Manyam GC (2014). Prevalence and clinical implications of epstein-barr virus infection in de novo diffuse large B-cell lymphoma in Western countries. Clin Cancer Res.

[5] Stuhlmann-Laeisz C, Borchert A, Quintanilla-Martinez L, Hoeller S, Tzankov A, Oschlies I (2016). In Europe expression of EBNA2 is associated with poor survival in EBV-positive diffuse large B-cell lymphoma of the elderly. Leuk Lymphoma.

[6] Ok CY, Papathomas TG, Medeiros LJ, Young KH (2013). EBV-positive diffuse large B-cell lymphoma of the elderly. Blood..

[7] Nicolae A, Pittaluga S, Abdullah S, Steinberg SM, Pham TA, Davies-Hill T (2015). EBV-positive large B-cell lymphomas in young patients: a nodal lymphoma with evidence for a tolerogenic immune environment. Blood..

[8] Park S, Lee J, Ko YH, Han A, Jun HJ, Lee SC (2007). The impact of Epstein-Barr virus status on clinical outcome in diffuse large B-cell lymphoma. Blood..

[9] Oyama T, Yamamoto K, Asano N, Oshiro A, Suzuki R, Kagami Y (2007). Age-related EBV-associated B-cell lymphoproliferative disorders constitute a distinct clinicopathologic group: a study of 96 patients. Clin Cancer Res.

[10] Sarkozy C, Hung SS, Chavez EA, Duns G, Takata K, Chong LC (2021). Mutational landscape of gray zone lymphoma. Blood..

[11] Gebauer N, Kunstner A, Ketzer J, Witte HM, Rausch T, Benes V (2021). Genomic insights into the pathogenesis of Epstein-Barr virus-associated diffuse large B-cell lymphoma by whole-genome and targeted amplicon sequencing. Blood. Cancer J.

[12] Zhou Y, Xu Z, Lin W, Duan Y, Lu C, Liu W (2019). Comprehensive genomic profiling of EBV-positive diffuse large B-cell lymphoma and the expression and clinicopathological correlations of some related genes. Front Oncol.

[13] Ott G, Ziepert M, Klapper W, Horn H, Szczepanowski M, Bernd HW (2010). Immunoblastic morphology but not the immunohistochemical GCB/nonGCB classifier predicts outcome in diffuse large B-cell lymphoma in the RICOVER-60 trial of the DSHNHL. Blood..

[14] Hanel P, Hummel M, Anagnostopoulos I, Stein H (2001). Analysis of single EBER-positive and negative tumour cells in EBV-harbouring B-cell non-Hodgkin lymphomas. J Pathol.

[15] Horn H, Ziepert M, Becher C, Barth TF, Bernd HW, Feller AC (2013). MYC status in concert with BCL2 and BCL6 expression predicts outcome in diffuse large B-cell lymphoma. Blood..

[16] Horn H, Staiger AM, Vohringer M, Hay U, Campo E, Rosenwald A (2015). Diffuse large B-cell lymphomas of immunoblastic type are a major reservoir for MYC-IGH translocations. Am J Surgical Pathol.

[17] Ventura RA, Martin-Subero JI, Jones M, McParland J, Gesk S, Mason DY (2006). FISH analysis for the detection of lymphoma-associated chromosomal abnormalities in routine paraffin-embedded tissue. J Mol Diagn.

[18] Hess G, Huttmann A, Witzens-Harig M, Dreyling MH, Keller U, Marks R, et al. A phase II trial to evaluate the combination of pixantrone and obinutuzumab for patients with relapsed aggressive lymphoma: Final results of the prospective, multicentre GOAL trial. Br J Haematol. 2022;198:482–91. 10.1111/bjh.18161 35362552

[19] Scott DW, Wright GW, Williams PM, Lih C-J, Walsh W, Jaffe ES, et al. Determining cell-of-origin subtypes of diffuse large B-cell lymphoma using gene expression in formalin-fixed paraffin embedded tissue. Blood. 2014;123:1214–7. 10.1182/blood-2013-11-536433 PMC3931191 24398326

[20] Scott DW, Mottok A, Ennishi D, Wright GW, Farinha P, Ben-Neriah S (2015). Prognostic significance of diffuse large B-cell lymphoma cell of origin determined by digital gene expression in formalin-fixed paraffin-embedded tissue biopsies. J Clin Oncol.

[21] Martincorena I, Raine KM, Gerstung M, Dawson KJ, Haase K, Van Loo P (2017). Universal patterns of selection in cancer and somatic tissues. Cell..

[22] Frontzek F, Staiger AM, Zapukhlyak M, Xu W, Bonzheim I, Borgmann V (2021). Molecular and functional profiling identifies therapeutically targetable vulnerabilities in plasmablastic lymphoma. Nat Commun.

[23] Van Loo P, Nordgard SH, Lingjaerde OC, Russnes HG, Rye IH, Sun W (2010). Allele-specific copy number analysis of tumors. Proc Natl Acad Sci USA.

[24] Mermel CH, Schumacher SE, Hill B, Meyerson ML, Beroukhim R, Getz G (2011). GISTIC2.0 facilitates sensitive and confident localization of the targets of focal somatic copy-number alteration in human cancers. Genome Biol.

[25] Wright GW, Huang DW, Phelan JD, Coulibaly ZA, Roulland S, Young RM (2020). A probabilistic classification tool for genetic subtypes of diffuse large B cell lymphoma with therapeutic implications. Cancer Cell.

[26] Schmitz R, Wright GW, Huang DW, Johnson CA, Phelan JD, Wang JQ (2018). Genetics and pathogenesis of diffuse large B-cell lymphoma. N. Engl J Med.

[27] Benjamini Y, Hochberg Y (1995). Controlling the false discovery rate - a practical and powerful approach to multiple testing. J R Stat Soc B.

[28] Rosenwald A, Bens S, Advani R, Barrans S, Copie-Bergman C, Elsensohn MH (2019). Prognostic significance of MYC rearrangement and translocation partner in diffuse large B-cell lymphoma: a study by the Lunenburg lymphoma biomarker consortium. J Clin Oncol.

[29] Liau NPD, Laktyushin A, Lucet IS, Murphy JM, Yao S, Whitlock E (2018). The molecular basis of JAK/STAT inhibition by SOCS1. Nat Commun.

[30] Alexander WS, Starr R, Fenner JE, Scott CL, Handman E, Sprigg NS (1999). SOCS1 is a critical inhibitor of interferon gamma signaling and prevents the potentially fatal neonatal actions of this cytokine. Cell..

[31] Rogers S, Wells R, Rechsteiner M (1986). Amino acid sequences common to rapidly degraded proteins: the PEST hypothesis. Science..

[32] Weng AP, Ferrando AA, Lee W (2004). Morris JPt, Silverman LB, Sanchez-Irizarry C, et al. Activating mutations of NOTCH1 in human T cell acute lymphoblastic leukemia. Science..

[33] Su YW, Hao Z, Hirao A, Yamamoto K, Lin WJ, Young A (2011). 14-3-3sigma regulates B-cell homeostasis through stabilization of FOXO1. Proc Natl Acad Sci USA.

[34] Trinh DL, Scott DW, Morin RD, Mendez-Lago M, An J, Jones SJ (2013). Analysis of FOXO1 mutations in diffuse large B-cell lymphoma. Blood..

[35] Zeytun A, Nagarkatti M, Nagarkatti PS (2000). Growth of FasL-bearing tumor cells in syngeneic murine host induces apoptosis and toxicity in Fas(+) organs. Blood..

[36] Prensner JR, Iyer MK, Sahu A, Asangani IA, Cao Q, Patel L (2013). The long noncoding RNA SChLAP1 promotes aggressive prostate cancer and antagonizes the SWI/SNF complex. Nat Genet.

[37] Veeck J, Dahl E (2012). Targeting the Wnt pathway in cancer: the emerging role of Dickkopf-3. Biochim Biophys Acta.

[38] Lacy SE, Barrans SL, Beer PA, Painter D, Smith AG, Roman E (2020). Targeted sequencing in DLBCL, molecular subtypes, and outcomes: a Haematological Malignancy Research Network report. Blood..

[39] Chapuy B, Stewart C, Dunford AJ, Kim J, Kamburov A, Redd RA (2018). Molecular subtypes of diffuse large B cell lymphoma are associated with distinct pathogenic mechanisms and outcomes. Nat Med.

[40] Montes-Moreno S, Odqvist L, Diaz-Perez JA, Lopez AB, de Villambrosia SG, Mazorra F (2012). EBV-positive diffuse large B-cell lymphoma of the elderly is an aggressive post-germinal center B-cell neoplasm characterized by prominent nuclear factor-kB activation. Mod Pathol.

[41] Kaye KM, Izumi KM, Kieff E (1993). Epstein-Barr virus latent membrane protein 1 is essential for B-lymphocyte growth transformation. Proc Natl Acad Sci USA.

[42] Uchida J, Yasui T, Takaoka-Shichijo Y, Muraoka M, Kulwichit W, Raab-Traub N (1999). Mimicry of CD40 signals by Epstein-Barr virus LMP1 in B lymphocyte responses. Science..

[43] Adam P, Bonzheim I, Fend F, Quintanilla-Martinez L (2011). Epstein-Barr virus-positive diffuse large B-cell lymphomas of the elderly. Adv Anat Pathol.

[44] Kataoka K, Miyoshi H, Sakata S, Dobashi A, Couronne L, Kogure Y (2019). Frequent structural variations involving programmed death ligands in Epstein-Barr virus-associated lymphomas. Leukemia..

[45] Gebauer N, Gebauer J, Hardel TT, Bernard V, Biersack H, Lehnert H (2015). Prevalence of targetable oncogenic mutations and genomic alterations in Epstein-Barr virus-associated diffuse large B-cell lymphoma of the elderly. Leuk Lymphoma.

[46] Gires O, Kohlhuber F, Kilger E, Baumann M, Kieser A, Kaiser C (1999). Latent membrane protein 1 of Epstein-Barr virus interacts with JAK3 and activates STAT proteins. EMBO J.

[47] Mottok A, Renne C, Seifert M, Oppermann E, Bechstein W, Hansmann ML (2009). Inactivating SOCS1 mutations are caused by aberrant somatic hypermutation and restricted to a subset of B-cell lymphoma entities. Blood..

[48] Chapman JR, Bouska AC, Zhang W, Alderuccio JP, Lossos IS, Rimsza LM (2021). EBV-positive HIV-associated diffuse large B cell lymphomas are characterized by JAK/STAT (STAT3) pathway mutations and unique clinicopathologic features. Br J Haematol.

[49] Shanmugam V, Craig JW, Hilton LK, Nguyen MH, Rushton CK, Fahimdanesh K (2021). Notch activation is pervasive in SMZL and uncommon in DLBCL: implications for Notch signaling in B-cell tumors. Blood Adv.

[50] Puente XS, Pinyol M, Quesada V, Conde L, Ordonez GR, Villamor N (2011). Whole-genome sequencing identifies recurrent mutations in chronic lymphocytic leukaemia. Nature..

[51] Bea S, Valdes-Mas R, Navarro A, Salaverria I, Martin-Garcia D, Jares P (2013). Landscape of somatic mutations and clonal evolution in mantle cell lymphoma. Proc Natl Acad Sci USA.

[52] Zimber-Strobl U, Strobl LJEBNA2 (2001). and Notch signalling in Epstein-Barr virus mediated immortalization of B lymphocytes. Semin Cancer Biol.

[53] Hofelmayr H, Strobl LJ, Marschall G, Bornkamm GW, Zimber-Strobl U (2001). Activated Notch1 can transiently substitute for EBNA2 in the maintenance of proliferation of LMP1-expressing immortalized B cells. J Virol.

[54] Challa-Malladi M, Lieu YK, Califano O, Holmes AB, Bhagat G, Murty VV (2011). Combined genetic inactivation of beta2-Microglobulin and CD58 reveals frequent escape from immune recognition in diffuse large B cell lymphoma. Cancer Cell.

[55] Choi IK, Wang Z, Ke Q, Hong M, Paul DW, Fernandes SM (2021). Mechanism of EBV inducing anti-tumour immunity and its therapeutic use. Nature..

[56] Polprasert C, Takeuchi Y, Makishima H, Wudhikarn K, Kakiuchi N, Tangnuntachai N (2021). Frequent mutations in HLA and related genes in extranodal NK/T cell lymphomas. Leuk Lymphoma.

[57] Li YY, Chung GT, Lui VW, To KF, Ma BB, Chow C (2017). Exome and genome sequencing of nasopharynx cancer identifies NF-kappaB pathway activating mutations. Nat Commun.

[58] Green MR, Monti S, Rodig SJ, Juszczynski P, Currie T, O’Donnell E (2010). Integrative analysis reveals selective 9p24.1 amplification, increased PD-1 ligand expression, and further induction via JAK2 in nodular sclerosing Hodgkin lymphoma and primary mediastinal large B-cell lymphoma. Blood..

[59] Chen BJ, Chapuy B, Ouyang J, Sun HH, Roemer MG, Xu ML (2013). PD-L1 expression is characteristic of a subset of aggressive B-cell lymphomas and virus-associated malignancies. Clin Cancer Res.

[60] Cancer Genome Atlas Research N. (2014). Comprehensive molecular characterization of gastric adenocarcinoma. Nature..

