# Supplementary material for: Molecular profiling of EBV associated diffuse large B-cell lymphoma
Source: Leukemia. 2023 Jan 5;37(3):670–9. doi: 10.1038/s41375-022-01804-w (PMC9991915; doi:10.1038/s41375-022-01804-w)
Supplement: Supplementary file 1 — Supplement Data [file 41375_2022_1804_MOESM1_ESM.pdf]

SUPPLEMENTARY DATA

Molecular profiling of EBV associated diffuse large B-cell lymphoma

Fabian Frontzek[1], Annette M. Staiger[2,3], Ramona Wullenkord[1], Michael Grau[1], Myroslav Zapukhlyak[1], Katrin S. Kurz[2], Heike Horn[2,3], Tabea Erdmann[1], Falko Fend[4], Julia Richter[5], Wolfram Klapper[5], Peter Lenz[6], Stephan Hailfinger[1], Anna Tasidou[7†], Marcel Trautmann[8], Wolfgang Hartmann[8], Andreas Rosenwald[9], Leticia Quintanilla-Martinez[4], German Ott[2,3], Ioannis Anagnostopoulos[9], Georg Lenz*[1]

[1]Department of Medicine A, Department of Hematology, Oncology and Pneumology, University Hospital Münster, Münster, Germany
[2]Department of Clinical Pathology, Robert Bosch Hospital, Stuttgart, Germany
[3]Dr. Margarete Fischer-Bosch Institute of Clinical Pharmacology, Stuttgart and University of Tuebingen, Germany
[4]Institute of Pathology and Neuropathology, Reference Center for Haematopathology University Hospital, Tübingen Eberhard-Karls-University, Tübingen, Germany
[5]Division of Hematophathology, Christian-Albrechts-University, Kiel, Germany
[6]Department of Physics, University of Marburg, Marburg, Germany
[7]Department of Hematopathology, Evangelismos General Hospital, Athens, Greece; †deceased in 2019
[8]Division of Translational Pathology, Gerhard-Domagk-Institute of Pathology, University Hospital Münster, Münster, Germany
[9]Institute of Pathology, University of Würzburg, Würzburg, Germany

Supplementary Methods

Targeted Sequencing and analysis of somatic DNA mutations

Library generation

We extracted 200 ng DNA per sample for targeted sequencing. DNA was sheared and ligated to specific adapters during automated library preparation using the Beckman FX^p liquid handling robot (SPRIworks, Beckman-Coulter, Pasadena, California, USA). Enrichment and capturing were performed applying the XT Fast Agilent SureSelect hybrid capture kit following manufacturer's instructions (Agilent Technologies, Santa Clara, California, USA).

Sequencing and preprocessing

Targeted deep sequencing was performed for 74 genes that were previously identified to be recurrently mutated in DLBCL. Sequencing was performed on a HiSeq platform (Illumina) with 250 bp paired-end reads. To align measured reads against the current human reference genome (GRCh38 version), we first extracted raw FASTQ reads from BAM formats with hg19 alignment utilizing the SamToFastq command of Picard tools version 2.25.0 (S1). Measured sequence reads were preprocessed and quality-controlled using cutadapt 3.2 (S2), Trim Galore! 0.6.6 (S3), and FastQC 0.11.9 (S4).

Sequence alignment

Trimmed reads were aligned against the current human reference genome from the Genome Reference Consortium (GRCh38) using HISAT2 v2.2.1 (S5), deduplicated using the MarkDuplicates command of Picard tools 2.25.0 (S6), and recalibrated by base score applying the Genome Analysis Toolkit v4.1.2.0 (GATK) (S7).

Variant discovery

We computed Mutect2 from the GATK to discover DNA variants (S8). Only reads aligned by HISAT2 that also passed GATK and Mutect quality control filters were further analyzed.

Basic variant filtering

To build a panel of normal variants (PON), we performed variant discovery with the same experimental and analytical pipeline for all normal controls (S9). Overall, we

sequenced extracted DNA of 22 unmatched normal tissues originating from healthy donors. A variant was included in the PON if determined to be significant in at least two independent subjects by Mutect. This PON was subsequently used to filter germline variants and potential systematic pipeline-specific artefacts (unpaired analysis mode). Additionally, we used the gnomAD database based on the Exome Aggregation Consortium ExAC (S10) as large population resource for filtering germline variants.

Variant annotation and advanced filtering

Next, we applied an optimized multistage filter hierarchy to reach maximal specificity of somatic mutation calls. All filter steps in the applied order are listed in Supplementary Table 3. For this hierarchy, we first annotated discovered variants with their transcript and protein level consequences using TransVar 2.4.1 (S11) and the NCBI RefSeq gene models (S12). In case of multiple RefSeq transcripts per gene, we annotated each variant with the one leading to the strongest possible biological consequence on protein level according to TransVar. For mutation overview plots, we selected the first principal transcript of the respective gene according to the APPRIS database (S13). Additionally, we annotated variants with confirmed somatic mutations according to the Catalogue Of Somatic Mutations In Cancer (COSMIC v85) (S9), the NCBI database of common human variants (≥5%) in any of the five large populations from dbSNP build 151 (S14), and NCBI ClinVar (version 2018-04) (S15) using vcfanno v0.3.0 (S16).

Somatic variants

Based on variant statistics from Mutect, GATK, and all annotations, our filter hierarchy called 0.43% of all variants in this targeted panel as somatic mutations, i.e. 4.83 variants on average per sample. See Supplementary Table 3 for detailed mutation counts and percentages remaining after each filtering step. All somatic mutations are provided in Supplementary Table 4.

Additional tools and software utilized for sequencing analysis

For various analytical tasks in the sequencing pipeline, we used bedtools 2.26.0 (S17), the Integrated Genomics Viewer 2.10.2 (S18), the Picard toolkit 2.25.0 (S1), and SAMtools 1.9 (S19). For analysis pipeline orchestration, including parallel remote analysis jobs on high performance clusters as well as for most visualizations including

oncoplots, we applied MATLAB® (version R2021a, The MathWorks® Inc., Natick, Massachusetts, USA), R (version 3.6.3-4.1.0, R Foundation for Statistical Computing, Vienna, Austria), Python (version 2.7-3.X, Python Software Foundation, Wilmington, Delaware, USA), and GNU Parallel (S20). Needle plots of mutation profiles were created using ProteinPaint (S21). All tools used, their versions, and their availabilities are summarized in Supplementary Table 8.

Frontzek et al. Supplementary Figure 1

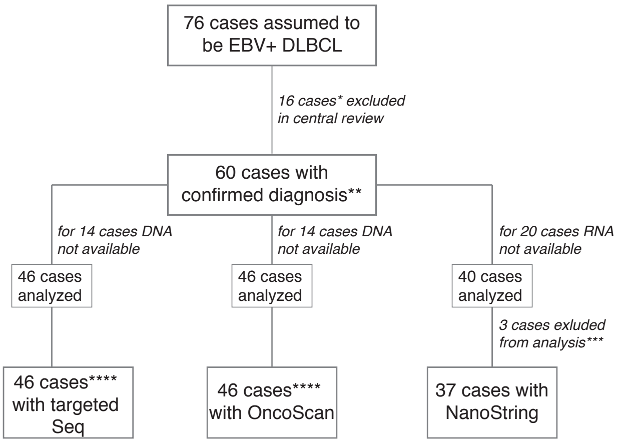

Supplementary Figure 1: All cases with assumed histology of EBV associated DLBCL NOS were centrally re-evaluated by three expert hematopathologists. *Overall, 16 cases had to be exluded due to the following reasons: EBER < 10% (n=2), diagnosis of angioimmunoblastic T-cell lymphoma (n=1), plasmablastic lymphoma (n=2), post-transplant lymphoproliferative disorder (n=1), plasmacytic differentiation (n=2), Hodgkin lymphoma (n=2), marginal zone lymphoma (n=1), other prior lymphoma diagnosis (n=3), and lacking expression of CD20 (n=2). **51 reviewed cases fulfilled all diagnostic criteria with more than 50% of lymphoma cells being positive in EBER. Nine further cases fulfilling all morphological criteria with EBER values <50% but >10% were added to our study cohort. ***Three cases showed borderline results in NanoString analysis and were subsequently excluded. ****The 46 out of 60 cases available for targeted DNA-sequencing and for OncoScan analysis were not identical (overlap of 40/60).

Frontzek et al. Supplementary Figure 2

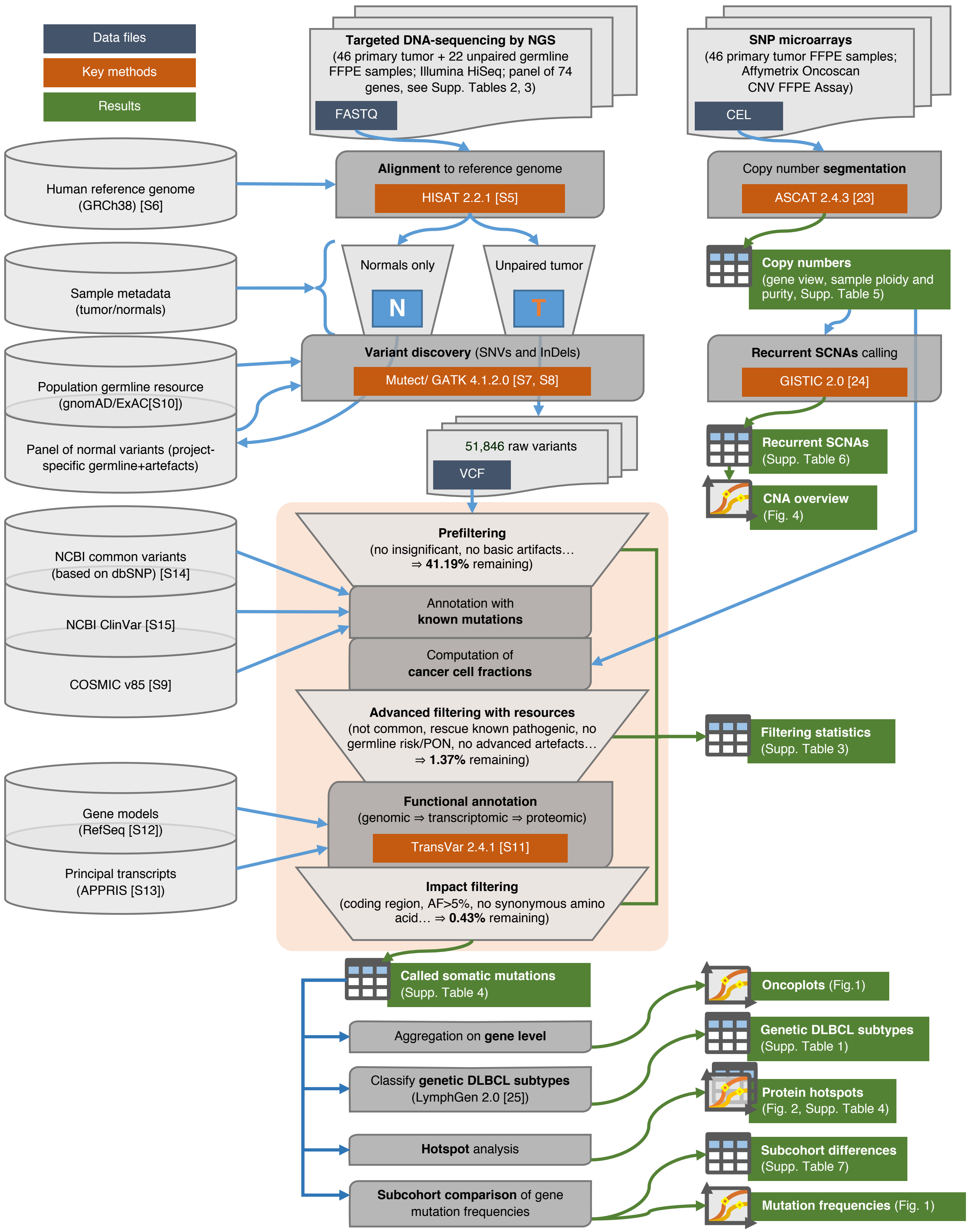

Supplementary Figure 2: Overview of the analytical work flow. Resource databases and metadata are depicted as grey discs, primary source data are shown in dark blue, key methods in orange, and resulting figures and tables in green color.

Frontzek et al. Supplementary Figure 3

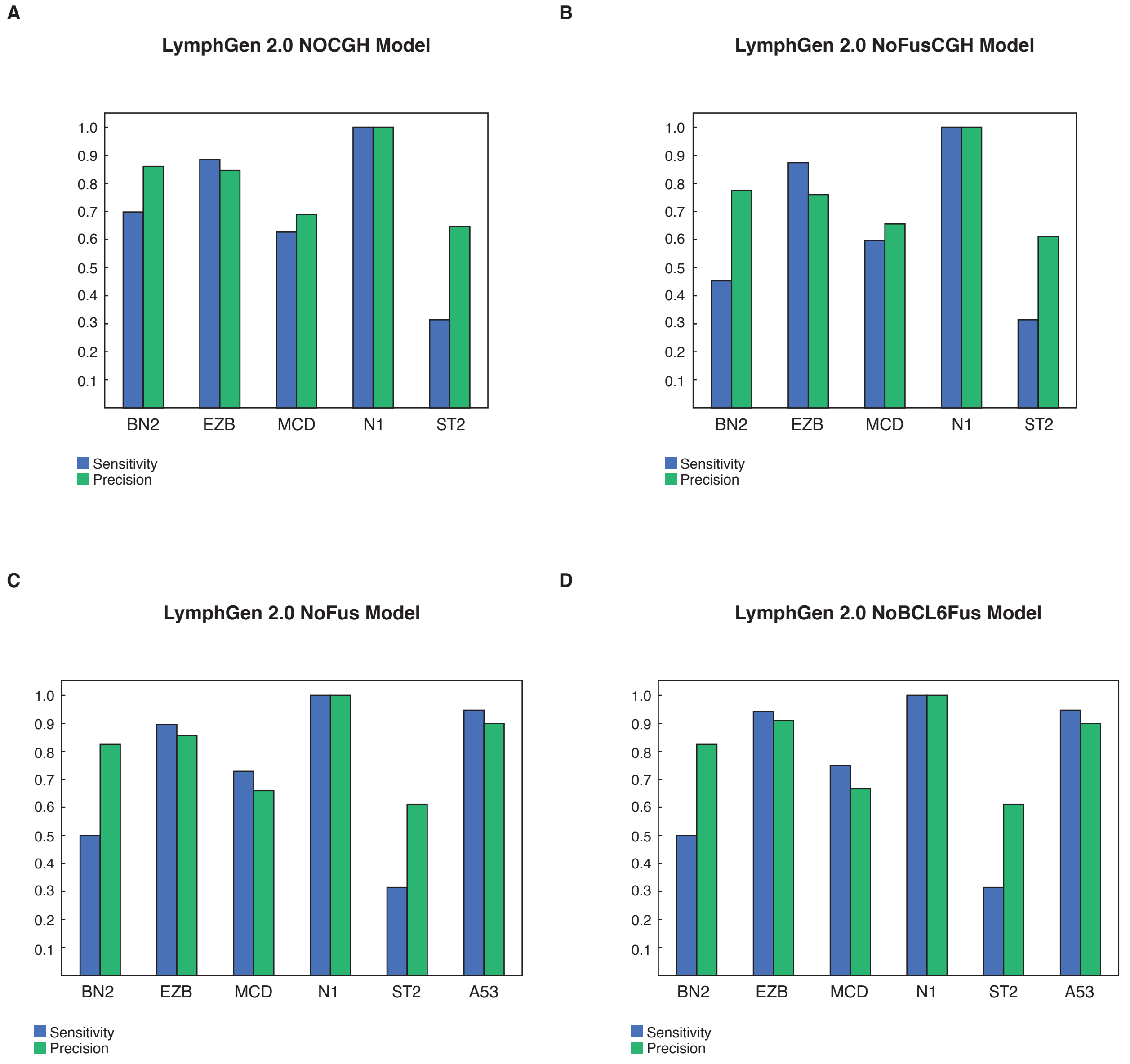

Supplementary Figure 3: Bar graph showing the sensitivity (blue) and precision (green) for prediction of molecular DLBCL subtypes in our cohort of EBV+ DLBCLs applying the **A** NOCGH Model, **B** NoFusCGH Model, **C** NoFus Model, and **D** the NoBCL6Fus Model according to the LymphGen classifier 2.0 (25).

Supplementary Table 2: Overview of target genes.

ARID1A	*MTOR*
ATM	*MYC*
B2M	*MYD88*
BCL2	*NFKBIA*
BCL6	*NOTCH1*
BCL10	*NOTCH2*
BIRC2	*PCLO*
BIRC3	*PIK3CD*
BRAF	*PIK3R1*
BTG1	*PLCG2*
BTG2	*PRDM1*
BTK	*PRKCB*
CARD11	*PTEN*
CCND3	*REL*
CD58	*SGK1*
CD79A	*SMARCA4*
CD79B	*SOCS1*
CREBBP	*STAT3*
CSNK1A1	*STAT6*
CYLD	*SYK*
EP300	*TAB2*
ERBB2	*TAB3*
EZH2	*TC7L1*
FOXO1	*TLR2*
GNA13	*TNF*
ID3	*TNFAIP3*
IRF8	*TNFRSF11A*
ITK	*TNFRSF13B*
JAK2	*TNFRSF13C*
KLHL6	*TNFRSF14*
KMT2D	*TNFSF13B*
LCK	*TP53*
LYN	*TANK*
MAP3K7	*TRAF3*
MAP3K14	*TRAF5*
MEF2B	*UBR5*
KMT2C	*NSD2*

Supplementary Table 8: Methods, tools, resources and software used in this study.

Method/Tool/Software	Version	Available at
HISAT2	2.2.1	http://daehwankimlab.github.io/hisat2/download
Genome Analysis Toolkit (GATK) / Mutect	4.1.2.0	https://github.com/broadinstitute/gatk/releases
TransVar	2.4.1	https://github.com/zwdzwd/transvar
LymphGen	2.0	https://llmpp.nih.gov/lymphgen/lymphgendataportal.php
dNdScv	0.1.0	https://github.com/im3sanger/dndscv
Chromosome Analysis Suite (ChAS)	4.3	https://www.thermofisher.com/chas
ASCAT	2.4.3	https://github.com/Crick-CancerGenomics/ascat
GISTIC	2.0	https://broadinstitute.github.io/gistic2/
Integrated Genomics Viewer	2.10.2	http://software.broadinstitute.org/software/igv/download
Protein Paint	n.a. (web app)	https://pecan.stjude.cloud/proteinpaint
GNU parallel	20161222	https://www.gnu.org/software/parallel/
samtools	1.9	http://www.htslib.org
bedtools	2.26.0	https://bedtools.readthedocs.io
Picard tools	2.25.0	https://broadinstitute.github.io/picard/
vcfanno	0.3.0	https://github.com/brentp/vcfanno/releases
Trim Galore!	0.6.6	https://github.com/FelixKrueger/TrimGalore
cutadapt	3.2	https://cutadapt.readthedocs.io/en/stable/
FastQC	0.11.9	http://www.bioinformatics.babraham.ac.uk/projects/fastqc
**Resources/Databases**	**Version**	**Available at**
COSMIC	85	https://cancer.sanger.ac.uk/cosmic
NCBI ClinVar	20180429	https://www.ncbi.nlm.nih.gov/clinvar/
gnomAD/ExAC	based on v2	Provided via GATK resource pack (af-only-gnomad.hg38.ensemble.vcf.gz); created from gnomAD by https://github.com/broadinstitute/gatk/blob/master/scripts/mutect2_wdl/mutect_resources.wdl
NCBI Common Human Variants	“common_all.vcf.gz” from 20180418	https://www.ncbi.nlm.nih.gov/variation/docs/human_variation_vcf
NCBI RefSeq gene models	20190227	Provided via TransVar download; file name hg38.refseq.gff.gz.transvardb; downloaded 20190227
APPRIS	20200122	https://appris.bioinfo.cnio.es/#/downloads
Human Reference Genome	GRCh38	http://daehwankimlab.github.io/hisat2/download/#h-sapiens
**IDEs and runtimes**	**Version**	**Available at**
MATLAB	R2021a	https://www.mathworks.com/pricing-licensing.html?prodcode=ML&intendeduse=edu
Python	2.7 and 3.6	https://www.python.org/
R	3.6.3 and 4.1.0	https://www.r-project.org/

Supplementary Table 9: Overview of genetic analyses of EBV+ DLBCLs.

	Gebauer et al. Blood Cancer J. 2021 (11)	Sarkozy et al. Blood 2021 (10)	Kataoka et al. Leukemia 2019 (44)	Frontzek et al. 2022
**Numbers of analyzed cases**	WGS/ CNA n=8<br>Target. seq n=47 (43 genes)	WES n=7<br>Target. seq n=13 (217 genes)	Target. seq/ CNA n=27 (140 genes, 1999 SNP probes)	Target. seq n=46 (74 genes)<br>OncoScan n=46
**EBER cut-off**	>50%	>90%	NA	>50% (n=51)<br>10-40% (n=9)
**Pathological central review**	Yes	Yes	NA	Yes
***Monomorphic vs. polymorphic* subtype**	Both	Polymorphic only	NA	Both
**JAK-STAT**	*STAT3:* NA<br>*STAT6:* 9%<br>*SOCS1:* 2%	*STAT3:* 15%<br>*STAT6:* 15%<br>*SOCS1:* 15%	*STAT3:* NA<br>*STAT6:* NA<br>*SOCS1:* NA	*STAT3:* 4%<br>*STAT6:* 2%<br>*SOCS1:* 24%
**NOTCH**	*NOTCH1*: NA<br>*NOTCH2*: 32%<br>*SPEN*: NA	*NOTCH1*: 5%<br>*NOTCH2*: 10%<br>*SPEN*: 15%	*NOTCH1*: NA<br>*NOTCH2*: NA<br>*SPEN*: NA	*NOTCH1*: 7%<br>*NOTCH2*: 15%<br>*SPEN*: NA
**Immune evasion**	*B2M*: 13%<br>*CD58*: 4%<br>*CIITA*: NA<br>*HLA-B*: NA	*B2M*: 5%<br>*CD58*: 15%<br>*CIITA*: 0%<br>*HLA-B*: 5%	*B2M*: 26%<br>*CD58*: 11%<br>*CIITA*: NA<br>*HLA-B*: 26%	*B2M*: 11%<br>*CD58*: 11%<br>*CIITA*: NA<br>*HLA-B*: NA
**NF-κB**	*CD79B:* 11%<br>*CARD11:* 9%<br>*MYD88:* 4%<br>*TNFAIP3:* NA	*CD79B:* 0%<br>*CARD11:* 0%<br>*MYD88:* 0%<br>*TNFAIP3*: 5%	*CD79B:* 0%<br>*CARD11:* 11%<br>*MYD88:* 4%<br>*TNFAIP3*: 4%	*CD79B:* 0%<br>*CARD11:* 2%<br>*MYD88:* 2%<br>*TNFAIP3*: 7%
**Epigenetic regulators**	*ARID1A:* 45%<br>*CREBBP:* 4%<br>*EZH2:* 9%<br>*KMT2A*: 32%<br>*KMT2C:* NA<br>*KMT2D:* 30%<br>*TET2:* NA	*ARID1A:* 5%<br>*CREBBP:* 0%<br>*EZH2:* 0%<br>*KMT2A*: 5%<br>*KMT2C:* 10%<br>*KMT2D:* 10%<br>*TET2:* 10%	*ARID1A*: NA<br>*CREBBP:* 11%<br>*EZH2:* 4%<br>*KMT2A*: NA<br>*KMT2C*: NA<br>*KMT2D:* 26%<br>*TET2:* 33%	*ARID1A*: 15%<br>*CREBBP:* 7%<br>*EZH2:* 4%<br>*KMT2A*: NA<br>*KMT2C*: 17%<br>*KMT2D:* 22%<br>*TET2:* NA
**Apoptosis/ Cell cycle/ DNA repair**	*ATM:* NA<br>*EP300*: NA<br>*FOXO1*: 2%<br>*TP53*: 9%	*ATM:* 5%<br>*EP300*: 10%<br>*FOXO1*: 5%<br>*TP53*: 5%	*ATM:* NA<br>*EP300*: 11%<br>*FOXO1*: NA<br>*TP53*: 26%	*ATM:* 7%<br>*EP300*: 13%<br>*FOXO1*: 13%<br>*TP53*: 7%

Abbreviations: WGS=whole genome sequencing, CNA=copy number analysis, target.=targeted, seq=sequencing, EBER= EBV-encoded small RNAs

References for Supplement

S1. Institute B. Picard Toolkit: a set of command line tools (in Java) for manipulating high-throughput sequencing (HTS) data and formats such as SAM/BAM/CRAM and VCF. . 2019.

S2. Martin M. Cutadapt removes adapter sequences from high-throughput sequencing reads. . EMBnetjournal. 2011(17(1), pp. 10-12).

S3. F. K. Trim Galore! is a wrapper script to automate quality and adapter trimming. 2012 [Available from: https://www.bioinformatics.babraham.ac.uk/projects/trim_galore/.

S4. Andrews S. FastQC: a quality control tool for high throughput sequence data. 2010 [Available from: http://www.bioinformatics.babraham.ac.uk/projects/fastqc.

S5. Kim D, Langmead B, Salzberg SL. HISAT: a fast spliced aligner with low memory requirements. Nature methods. 2015;12(4):357-60.

S6. Schneider VA, Graves-Lindsay T, Howe K, Bouk N, Chen HC, Kitts PA, et al. Evaluation of GRCh38 and de novo haploid genome assemblies demonstrates the enduring quality of the reference assembly. Genome research. 2017;27(5):849-64.

S7. McKenna A, Hanna M, Banks E, Sivachenko A, Cibulskis K, Kernytsky A, et al. The Genome Analysis Toolkit: a MapReduce framework for analyzing next-generation DNA sequencing data. Genome research. 2010;20(9):1297-303.

S8. Cibulskis K, Lawrence MS, Carter SL, Sivachenko A, Jaffe D, Sougnez C, et al. Sensitive detection of somatic point mutations in impure and heterogeneous cancer samples. Nature biotechnology. 2013;31(3):213-9.

S9. Tate JG, Bamford S, Jubb HC, Sondka Z, Beare DM, Bindal N, et al. COSMIC: the Catalogue Of Somatic Mutations In Cancer. Nucleic acids research. 2019;47(D1):D941-D7.

S10. Lek M, Karczewski KJ, Minikel EV, Samocha KE, Banks E, Fennell T, et al. Analysis of protein-coding genetic variation in 60,706 humans. Nature. 2016;536(7616):285-91.

S11. Zhou W, Chen T, Chong Z, Rohrdanz MA, Melott JM, Wakefield C, et al. TransVar: a multilevel variant annotator for precision genomics. Nature methods. 2015;12(11):1002-3.

S12. O'Leary NA, Wright MW, Brister JR, Ciufo S, Haddad D, McVeigh R, et al. Reference sequence (RefSeq) database at NCBI: current status, taxonomic expansion, and functional annotation. Nucleic acids research. 2016;44(D1):D733-45.

S13. Rodriguez JM, Maietta P, Ezkurdia I, Pietrelli A, Wesselink JJ, Lopez G, et al. APPRIS: annotation of principal and alternative splice isoforms. Nucleic acids research. 2013;41(Database issue):D110-7.

S14. Sherry ST, Ward MH, Kholodov M, Baker J, Phan L, Smigielski EM, et al. dbSNP: the NCBI database of genetic variation. Nucleic acids research. 2001;29(1):308-11.

S15. Landrum MJ, Lee JM, Benson M, Brown GR, Chao C, Chitipiralla S, et al. ClinVar: improving access to variant interpretations and supporting evidence. Nucleic acids research. 2018;46(D1):D1062-D7.

S16. Pedersen BS, Layer RM, Quinlan AR. Vcfanno: fast, flexible annotation of genetic variants. Genome biology. 2016;17(1):118.

S17. Quinlan AR, Hall IM. BEDTools: a flexible suite of utilities for comparing genomic features. Bioinformatics. 2010;26(6):841-2.

S18. Thorvaldsdottir H, Robinson JT, Mesirov JP. Integrative Genomics Viewer (IGV): high-performance genomics data visualization and exploration. Briefings in bioinformatics. 2013;14(2):178-92.

S19. Li H, Handsaker B, Wysoker A, Fennell T, Ruan J, Homer N, et al. The Sequence Alignment/Map format and SAMtools. Bioinformatics. 2009;25(16):2078-9.

S20. Tange O. GNU Parallel - The Command-Line Power Tool. The USENIX Magazine. 2011;42-47.

S21. Zhou X, Edmonson MN, Wilkinson MR, Patel A, Wu G, Liu Y, et al. Exploring genomic alteration in pediatric cancer using ProteinPaint. Nat Genet. 2016;48(1):4-6.
